# Treg and CTLA-4: Two intertwining pathways to immune tolerance

**DOI:** 10.1016/j.jaut.2013.06.006

**Published:** 2013-09

**Authors:** Lucy S.K. Walker

**Affiliations:** Institute of Immunity & Transplantation, University College London Medical School, Royal Free Campus, Rowland Hill Street, London NW3 2PF, UK

**Keywords:** CTLA-4, Treg, Foxp3, Immune regulation, CD4 T cells, Tolerance

## Abstract

Both the CTLA-4 pathway and regulatory T cells (Treg) are essential for the control of immune homeostasis. Their therapeutic relevance is highlighted by the increasing use of anti-CTLA-4 antibody in tumor therapy and the development of Treg cell transfer strategies for use in autoimmunity and transplantation settings. The CTLA-4 pathway first came to the attention of the immunological community in 1995 with the discovery that mice deficient in *Ctla-4* suffered a fatal lymphoproliferative syndrome. Eight years later, mice lacking the critical Treg transcription factor *Foxp3* were shown to exhibit a remarkably similar phenotype. Much of the debate since has centered on the question of whether Treg suppressive function requires CTLA-4. The finding that it does in some settings but not in others has provoked controversy and inevitable polarization of opinion. In this article, I suggest that CTLA-4 and Treg represent complementary and largely overlapping mechanisms of immune tolerance. I argue that Treg commonly use CTLA-4 to effect suppression, however CTLA-4 can also function in the non-Treg compartment while Treg can invoke CTLA-4-independent mechanisms of suppression. The notion that Foxp3 and CTLA-4 direct independent programs of immune regulation, which in practice overlap to a significant extent, will hopefully help move us towards a better appreciation of the underlying biology and therapeutic significance of these pathways.

## Highlights

• CTLA-4 and Treg emerged independently as critical regulators of immune homeostasis.
• The history and biological role of each pathway is discussed.
• The extent and nature of the overlap between the two pathways is considered.

## 1 Introduction

Despite the selection processes applied to T cells during their development in the thymus, T cells with the capacity to recognize self-proteins nevertheless arise and populate the periphery. These T cells have the potential to cause autoimmune diseases, but generally do not because powerful peripheral tolerance mechanisms keep them in check [1]. The archetypal example of peripheral tolerance is provided by regulatory T cells (Treg) that are endowed with potent immunosuppressive capacity and whose continued presence is essential to prevent catastrophic over-activation of the immune system [2]. Equally critical for immune regulation is the T cell inhibitory protein CTLA-4, deficiency of which triggers lethal autoimmunity [3,4]. Although the CTLA-4 and Treg fields emerged independently, recent years have witnessed a striking convergence of the two research areas. This article seeks to present the historical context of these two areas, explore their intersection and finally highlight some of the pertinent questions remaining in this field.

## 2 Control of T cell responses by CTLA-4

### 2.1 Costimulatory control of T cell activation

**Fig. 1:**
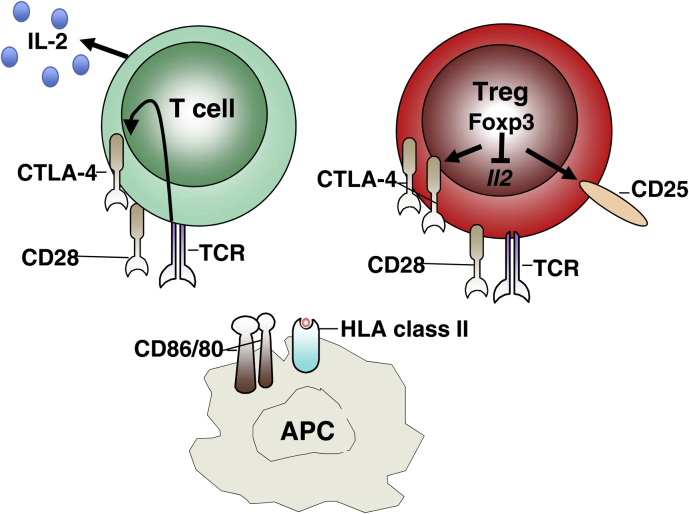
Cellular expression of CTLA-4 and Foxp3. CTLA-4 is a predominantly intracellular protein that is constitutively expressed in Foxp3+ Treg and induced in conventional T cells following activation. CTLA-4 and CD28 bind to shared ligands (CD80, CD86) on antigen presenting cells. The Treg transcription factor, Foxp3, promotes expression of CTLA-4 and other characteristicTreg markers such as CD25 while inhibiting expression of cytokines such as IL-2.

CTLA-4 is an inhibitory relative of the T cell costimulatory molecule CD28. While CD28 signaling promotes T cell activation, CTLA-4 serves an immunoregulatory function, suppressing the T cell response [5]. Despite their opposing functions, both CD28 and CTLA-4 interact with the same shared ligands CD80 (B7.1) and CD86 (B7.2)(reviewed in Ref. [6]). The superior affinity of CTLA-4 for both ligands [7] is balanced by its predominantly intracellular location [8] contrasting with CD28 which is constitutively expressed at the cell surface (see Fig. 1.). A diverse array of mechanisms has been proposed to account for the inhibitory function of CTLA-4; these include competing with CD28 for binding to their shared ligands, downregulating ligand expression and transmitting inhibitory signals. The molecular mechanisms of CTLA-4 function are not the focus of this article and are extensively reviewed elsewhere [9–13].

### 2.2 CTLA-4: a critical inhibitor of autoimmunity

Widespread recognition of the importance of the CTLA-4 pathway came about when mice deficient in the *Ctla4* gene were found to exhibit dysregulated T cell immunity resulting in tissue infiltration and death around 3wk of age [3,4]. Pathology resulted from the unchecked expansion of T cells possessing a diverse and unbiased TCR repertoire [14] and exhibiting reactivity against self tissues. Disease appeared to be driven by the CD4 compartment since depletion of CD4 T cells from birth effectively prevented lymphadenopathy and tissue infiltration [15]. A large body of subsequent work has confirmed the CTLA-4 pathway as a key arbiter in the choice between immunity and tolerance. Blockade of CTLA-4 with antibodies was shown to exacerbate disease in various mouse models of autoimmunity [16–18] and could even induce autoimmune manifestations in normal mice including gastritis, oophoritis, and mild sialoadenitis [19].

Consistent with the above observations, polymorphisms in the *Ctla4* locus have long been associated with autoimmunity [20–22] and further variation within the same gene cluster (CD28, ICOS) is likely to contribute to the net phenotype imparted by this region [23]. Several isoforms of CTLA-4 exist [21,24–29] and their relative expression levels may also influence CTLA-4-dependent immune regulation.

### 2.3 CTLA-4 regulates the CD28 pathway

Several lines of evidence support the view that the biological function of CTLA-4 is to control CD28 signaling. Blocking CD80 and CD86 with CTLA-4-Ig (thereby abrogating CD28 signaling) is known to inhibit disease in *Ctla4*−/− mice [30,31]. Similarly mice that lack CD80 and CD86 as well as CTLA-4 (i.e. triple knockout mice) show no signs of the immune dysregulation associated with CTLA-4 deficiency [32,33]. The requirement for CD80/CD86 to drive disease in *Ctla4*−/− mice reflects their engagement of CD28, since mice lacking both CD28 and CTLA-4 have no evidence of spontaneous T cell activation and do not develop pathology [34]. An intriguing study from the Singer laboratory delved deeper into this issue by probing which regions of the CD28 cytoplasmic domain were required for pathology in *Ctla4*−/− mice [35]. The candidate regions under investigation were the “YMNM” motif (residues 170–173) known to bind phosphoinositide 3-kinase (PI3K), Grb2 and Gads; the “N-terminal proline” motif “PRRP” (residues 175–178) that binds Itk; and the “C-terminal proline motif “PYAP” (residues 187–190) that associates with Lck, Fyn and Grb2. Strikingly this approach revealed that disease mapped to the status of the CD28 C-terminal proline motif. *Ctla4*−/− mice expressing CD28 molecules with two single point mutations in this motif remained completely healthy, whereas mutations in other regions of the CD28 cytoplasmic domain did not interrupt pathology. Collectively these studies provide strong evidence that the role of CTLA-4 is to regulate CD28-dependent T cell activation.

## 3 Control of T cell responses by Treg

### 3.1 Treg as essential immune regulators

The notion that the peripheral immune compartment is not entirely self tolerant but is policed by cells with regulatory activity has now been firmly incorporated into mainstream immunology. Early work by the Powrie, Sakaguchi and Shevach groups showed that cells with immunoregulatory activity could be identified on the basis of their CD45 isoform usage [36], or their expression of CD38 [37] or CD25 [38–41]. It is now well established that Treg are essential for the maintenance of tolerance to self-tissues (particularly those that announce their presence via secretory function) as well as regulating responses to environmental antigens, tumor antigens and infectious agents [42–44]. Treg have also been implicated in maintaining tolerance to the fetus during pregnancy [45–48] and the role of peripherally induced Treg may be particularly significant in this context [49]. Multiple suppressive mechanisms can be invoked by Treg [50,51] permitting them to control a broad range of target cell populations in different contexts.

### 3.2 Identification of the Treg transcription factor Foxp3

A key landmark in the Treg field was the identification of the transcription factor Foxp3 that plays a central role in directing the regulatory program (see Fig. 1). This discovery arose from a sequencing project [52] to determine the causal mutation in the scurfy mouse, an animal presenting with a severe lymphoproliferative syndrome [53]. The *Foxp3* gene was pinpointed as the culprit, and it was shown that a frameshift mutation in scurfy mice resulted in a product lacking the carboxy-terminal forkhead domain [52]. Crucially, the Sakaguchi [54], Rudensky [55] and Ramsdell [56] groups then made the link between the CD25+ Treg population and the immune-regulatory function of the *Foxp3* gene. It was demonstrated that Foxp3 expression was essentially confined to CD4+CD25+ cells and was responsible for the regulatory activity of this subset. Accordingly, adoptive transfer of CD4+CD25+ T cells from wildtype mice could rescue the lymphoproliferative syndrome in scurfy mice [55] and retroviral expression of *Foxp3* in CD25− T cells was shown to endow them with regulatory function [54,55]. Similarly, transgenic expression of *Foxp3* permitted CD25− T cells, and even CD8 T cells to acquire regulatory activity [56]. Consistent with the large body of evidence obtained in mouse models, mutations in the *Foxp3* gene in humans are associated with defective immune regulation, manifesting as a syndrome that has been termed immune dysregulation polyendocrinopathy enteropathy X-lined (IPEX) [57,58]. It is now well established that although some features of the Treg program emerge prior to [59] or independently of [60] Foxp3 expression, Foxp3 is nonetheless critical for enforcing the regulatory phenotype. In thymic-derived Treg, Foxp3 is turned on in developing thymocytes with the majority of Foxp3+ cells being CD4+CD8− cells and residing in the medulla [61]. The strength of TCR signaling, “translated” by induction of Nr4a nuclear receptors [62] and CD28 co-stimulation [63] both contribute to upregulation of Foxp3 intrathymically. However, expression of Foxp3 in the thymus alone is insufficient to prevent disease in scurfy mice [64] and ablation of Foxp3-expressing cells in adult mice (by exploiting Foxp3-driven diphtheria receptor expression) causes fatal autoimmunity [2], consistent with a requirement for continuous Foxp3 expression for Treg function. Treg preferentially accumulate in lymph nodes draining the tissues that express their cognate self-antigen [65] and as a consequence the Treg repertoire can vary considerably between different anatomical locations [66].

### 3.3 What does Foxp3 do?

The question of precisely what Foxp3 does to elicit the regulatory program has proved harder than expected to tease out. Analysis of Foxp3-bound genes has uncovered numerous targets [67,68], however none fulfilled the “holy grail” criterion of providing a convincing molecular explanation for Treg function. Indeed the Foxp3 target genes appeared to comprise only around 6% of the Foxp3-dependent genetic program [67]. Recent analysis suggests part of this puzzle may be explained by the ability of Foxp3 to associate with a surprisingly large number of co-factors that may broaden its functional potential. By fishing with a biotin-tagged Foxp3 protein in a T cell hybridoma, Rudra et al. were able to pull out no less than 361 binding partners for Foxp3 [69]. Interestingly, although these included some of the Foxp3-interacting proteins that had previously been described (e.g. NFAT [70] Runx [71]), other known binding-partners (e.g. Irf4 [72], Hif1a [73]) were not identified in this approach. This hints that contextual cues (e.g. activation stimuli, hypoxia) may influence the composition of the proteins recruited to the Foxp3 complex [69]. Thus the transcriptional program directed by Foxp3 is likely to depend on the cellular environment in which it is expressed since this will dictate its interactions with different co-factors. As a basic principle, this may explain why historically not all attempts to confer regulatory function by Foxp3 transduction have been successful [74,75] and why B cells from Foxp3 transgenic mice fail to exhibit suppressive function [56].

Intriguingly, a distinct subset of Foxp3-binding transcription factors appears to play a particularly important role in supporting its function. Fu and colleagues identified a “quintet” of transcription factors (Eos, IRF4, GATA-1, Lef1 and Satb1) that appear to reinforce the regulatory program by promoting Foxp3 occupancy of target sites and enhancing its transcriptional activity [76]. In human Treg, a novel Foxp3 interacting protein termed FIK (Foxp3-interacting KRAB domain-containing protein) has recently been identified that serves to couple Foxp3 with the chromatin-remodeling scaffold protein KAP1 [77]. The interaction of Foxp3 with FIK and KAP1 was found to be particularly important for its ability to downregulate genes such as IL-2 and IFNγ while it was not required for Foxp3-dependent upregulation of CTLA-4 and CD25. Collectively these studies suggest that molecular control of Foxp3-dependent regulation is highly complex involving large numbers of interacting cofactors and the capacity to integrate multiple contextual cues.

## 4 Intersection between Treg- and CTLA-4-mediated tolerance

### 4.1 Early convergence of the Treg and CTLA-4 fields

The similarity between the phenotype of *CTLA-4*-deficient and *Foxp3*-deficient mice sparked immediate interest in whether these two genes might function in a common pathway. In other words, could the CTLA-4 pathway explain the regulatory function of Foxp3+ Treg? Early indications of the overlap between CTLA-4 and Treg came from careful analysis of the regulation of CTLA-4 expression during T cell responses to peptide *in vivo* [78]. In this study Metzler and colleagues identified a small population of CTLA-4+ cells in mice that had not been immunized with specific peptide. They characterized this population as CD25+ and CD45RB^low^ and speculated that CTLA-4 expression might represent a “common denominator” underpinning the regulatory function of both CD25+ cells and CD45RB^low^ cells [78].

Potential links between the Foxp3 and CTLA-4 programs were also probed by testing whether CTLA-4 was absent from scurfy mice or whether CTLA-4-deficient animals lacked Treg. In both cases, quite the opposite was observed; scurfy [52] or Foxp3-deficient [55] mice expressed at least as much CTLA-4, if not more, than their wildtype counterparts and CTLA-4-deficient mice actually harbored an augmented Treg population [79–81]. Thus, despite the tantalizing similarity between the phenotypes of mice lacking either gene, Foxp3 was not required for CTLA-4 induction and nor was CTLA-4 required for expression of Foxp3.

### 4.2 Foxp3−/− and CTLA-4−/− phenotypes are corrected in the presence of wildtype cells

Consistent with the ability of Foxp3+ cells to elicit dominant regulation, the presence of wildtype cells has been shown to abolish the scurfy phenotype in mixed bone marrow chimeras [82,83]. In fact injection of 10^6^ CD4+CD25+ cells was able to prevent disease in Rag-deficient recipients of scurfy bone marrow [84] while transfer of 4 × 10^5^ wildtype CD4+CD25+ cells directly into 1–2 day old scurfy mice can restore immune homeostasis [55]. As expected, bone marrow cells from animals impaired in their capacity to generate Treg, as a result of Foxo1/Foxo3 deficiency, are unable to prevent the scurfy phenotype in mixed chimeras [83].

The correction of Treg deficiency (i.e. the scurfy phenotype) by the presence of wildtype cells could be viewed as entirely predictable, given the acknowledged role of this subset in eliciting cell-extrinsic regulation. Far more surprising was the observation that CTLA-4 deficiency could be compensated for in exactly the same way, namely by the mixing of CTLA-4−/− bone marrow with wildtype bone marrow in chimeric mice (see Table 1). This observation was reported by Bachmann and colleagues who found that rag−/− mice reconstituted with CTLA-4−/− bone marrow alone died roughly 10 weeks later whereas those that also received wildtype bone marrow remained completely healthy [85]. Even more striking was the finding that the CTLA-4−/− T cells in such mixed chimeras showed no signs of activation, suggesting that CTLA-4 expression on one T cell was able to control the activation status of another T cell. Given that an inhibitory signal delivered via the CTLA-4 receptor was then the favored model for CTLA-4 function, this result was initially baffling. However, the bone marrow chimera experiment has stood the test of time, proving remarkably reproducible in the hands of numerous investigators [33,82,86–88]. The simplest interpretation of these data is that CTLA-4 can function cell-extrinsically; i.e. T cells do not themselves need to express CTLA-4 to feel its force.

Given the ability of both CTLA-4+ cells and Foxp3+ cells to elicit dominant regulation in bone marrow chimeric mice, the Bluestone group sought to determine whether these two genes had to be expressed in the same cell for regulation to occur [82]. To this end, bone marrow from scurfy mice or CTLA-4−/− mice (also lacking CD80 and CD86) was injected alone or as a 1:1 mix into rag−/− recipients. For the first 50 days after transfer, the survival curves were indistinguishable between the 3 groups, with around 50% mortality during this period. However, subsequently a fraction of the animals receiving the mixed bone marrow showed a delayed decline, even though they all eventually died. These data hint at the ability of either Foxp3 or CTLA-4 (or both) to function independently of one another. However the fact that all mice receiving the mix of scurfy and CTLA-4−/− bone marrow died indicates that CTLA-4 and Foxp3 must be co-expressed in the same cell for efficient immune regulation to ensue. These data nicely complement the observation that transgenic overexpression of Foxp3 can delay lethality in CTLA-4−/− mice, but can only provide a temporary reprieve [56]. Together the data strongly suggest that effective immune regulation requires at least a subset of cells to co-express Foxp3 and CTLA-4.

### 4.3 Role for CTLA-4 in Treg function

Studies by the Powrie [89] and Sakaguchi [19] groups were the first to provide direct evidence that the CTLA-4 pathway could be used to elicit Treg suppression. However, the field was fraught with conflicting data. Early work from the Shevach group suggested that anti-CTLA-4 antibody failed to reverse Treg suppression *in vitro* [41], and others generated similar data [90]. A subsequent follow-up analysis by the Shevach group concluded that different preparations of anti-CTLA-4 antibody showed significant variation in their capacity to inhibit suppression [91]. In addition, interpretation of the results was complicated by the ability of such antibodies to augment conventional T cell proliferation [91], raising the possibility that the treatment increased conventional T cell (Tconv) proliferation rather than altering the extent of Treg suppression. This caveat was elegantly side-stepped in two other studies where anti-CTLA-4 Fab fragments were demonstrated to abolish suppression even in settings in which the conventional T cells were derived from CTLA-4−/− mice (and therefore could not be the target of the anti-CTLA-4 antibody) [19,31].

At face value, the above findings would appear to conclusively demonstrate a critical role for CTLA-4 in Treg function *in vitro*. However, this is not the whole story since testing this hypothesis by gene-deficiency has continued to produce conflicting results. Some studies show clearly that CTLA-4 deficiency abrogates Treg function in *in vitro* assays [81,88]. On the other hand, numerous reports have demonstrated that Treg from CTLA-4−/− mice retain suppressive function *in vitro* [31,79,92–94]. In some cases the CTLA-4−/− Treg suppress marginally less efficiently than wildtype Treg [79,93] mirroring the early observation that CTLA-4−/− Treg showed ∼50% suppression compared to the ∼95% suppression elicited by their wildtype counterparts [19]. Interestingly, Tang and colleagues found that even though CTLA-4−/− Treg were capable of suppression, the function of wildtype Treg was abrogated by anti-CTLA-4 antibody. This suggests that wildtype Treg use CTLA-4 to suppress but that compensatory mechanisms might develop in animals genetically deficient in the CTLA-4 pathway. The potential for gene-deficient animals to invoke compensatory pathways is elegantly illustrated by the observation that dual deficiency in IL-10 and IL-35 results in a striking compensatory increase in TRAIL (*tnfsf10*) expression and increased reliance on the TRAIL pathway for *in vitro* suppression [95].

The discrepancies in CTLA-4 dependence of Treg suppression *in vitro* hold true in human as well as mouse. For example in some studies anti-CTLA-4 antibody failed to interrupt human Treg suppression [96], while in others suppression was found to be largely CTLA-4 dependent with a minor contribution from TGFβ [97]. Supporting a role for CTLA-4 on Treg, depletion of CD25+ cells was shown to abrogate the ability of anti-CTLA-4 antibody to augment the proliferation of human T cells [98]. Thus, just like the analysis of mouse Treg, evidence both for and against a role for CTLA-4 in Treg function has been obtained *in vitro* using human cells. The most likely explanation for these conflicting data is that variation in assay conditions (APC type, strength of TCR stimulus... etc) has a profound impact on the CTLA-4 dependency of suppression. In at least two studies, CTLA-4−/− Treg were demonstrated to work *in vitro* yet lacked suppressive capacity *in vivo* [79,94] emphasizing the potential limitations of *in vitro* suppressive assays; ultimately their reductionist nature might permit redundant mechanisms to compensate for CTLA-4 deficiency more readily than in more complex *in vivo* situations.

There is now overwhelming evidence to support a role for CTLA-4 in the function of Treg in *in vivo* settings. A selection of this evidence is presented in Table 2. The emergence of cell-extrinsic models of CTLA-4 function [11–13] has provided a mechanistic basis for its role in the Treg compartment and a conceptual framework for interpretation of the original bone marrow chimera experiments. A key piece in this puzzle has been provided by the demonstration that CTLA-4 can physically remove its ligands from antigen presenting cells by a process of trans-endocytosis, affording a mechanism for Treg to regulate CD28 stimulation of other T cells [99].

Experimental settings in which CTLA-4-deficient Treg retain suppressive function *in vivo* can also be found in the literature [29,33,92] and serve as important reminders that alternative mechanisms can compensate for a lack of CTLA-4 in certain circumstances. Overall, the data point to a key role for CTLA-4 in Treg function although other mechanisms can sometimes substitute in its absence.

### 4.4 Role for CTLA-4 in the conventional T cell compartment

It has been clearly established that CTLA-4 can also function to regulate T cell responses when expressed on conventional T cells. Ironically, the general acceptance of this idea owes much to the early experiments showing that CTLA-4 antibodies altered T cell proliferation *in vitro* [100–103] that were performed prior to widespread recognition of the Treg lineage. These experiments were generally performed on whole CD4 T cells, rather than on purified CD4+CD25− cells; with the benefit of hindsight, it is likely that many of the CTLA-4 effects demonstrated in these early studies were actually a result of targeting CTLA-4 on the Treg population. Nevertheless, even when TCR transgenic systems have been used in subsequent studies to rigorously select against the presence of Treg it is clear that CTLA-4 can still function to regulate the magnitude of conventional T cell responses. Extensive evidence supports the function of CTLA-4 in the Tconv compartment [104–111], including the demonstration that CTLA-4 regulates the Tconv response to soluble antigen [104] and to tissue-derived neo-self antigen [105] as well as modulating the capacity of Tconv to infiltrate antigen-bearing tissues and cause destruction [108]. The finding that mice lacking CTLA-4 only in Treg live longer than those lacking CTLA-4 systemically [81] also points to a functional role for CTLA-4 in the conventional T cell population.

## 5 Overall perspectives

### 5.1 Why is Treg function CTLA-4-independent in some studies?

The demonstration that loss of CTLA-4 in the Treg compartment is sufficient to precipitate lethal autoimmunity [81] implies that CTLA-4 is of fundamental importance in the Treg lineage. That being so, it prompts the question; why don't investigators uniformly identify a role for CTLA-4 in Treg function? Although numerous studies report CTLA-4-dependent Treg function (see Table 2), there are notable exceptions where Treg lacking CTLA-4 elicit effective suppression. In my view, there are three main issues to consider in this regard; whether the response being targeted is CD28-dependent, the tissue site and differentiation state of the cells being regulated, and the nature of the Treg itself.

As emphasized earlier, the central biological role of CTLA-4 is to regulate the CD28 pathway (potentially via ligand competition [112] and ligand downregulation [99,113–115] although other mechanisms are also plausible). It follows that T cell responses that do not utilize CD28 costimulation would not be predicted to be subject to CTLA-4-dependent regulation. Interestingly, a recent study using BDC2.5 Treg found that CTLA-4-deficient cells were just as effective as their wildtype counterparts at regulating diabetes in an adoptive transfer model [29]. However, the diabetes model employed was the CD28KO.NOD mouse in which the diabetogenic T cell response is, by definition, CD28-independent. Thus, the failure to demonstrate CTLA-4-dependent immune regulation in this setting is expected, given that competition for (or downregulation of) CD86 and CD80 would not be predicted to alter the activation of CD28-deficient T cells. In contrast, in a different TCR-transgenic diabetes model, CTLA-4 deficiency abrogated the ability of Treg to control disease [79]. The latter model utilized CD28-sufficient mice and the diabetogenic T cell response is CD28-dependent in this system (Wang and Walker, unpublished observation) providing a potential explanation for this difference.

Regarding context of the response being regulated, susceptibility to regulation via non-CTLA-4-based mechanisms may vary depending on the tissue site and differentiation state of the T cells. In the gut, IL-10 production may represent a good alternative to CTLA-4 in controlling errant T cell responses. Accordingly regulation of colitis by wildtype Treg can be blocked by anti-CTLA-4 antibody, but CTLA-4-deficient Treg can instead utilize IL-10 to achieve regulation [33]. In the pancreas, on the other hand, IL-10 may be a less reliable inhibitor of T cell immunity. While IL-10 can inhibit diabetes under certain circumstances [116,117], transgenic expression of IL-10 in the pancreas can actually exacerbate diabetes [118]. Thus, IL-10 produced by CTLA-4-deficient Tregs could conceivably be less effective at eliciting immunosuppression in the pancreas. The stage of the immune response may also dictate the relative efficacy of different suppressive mechanisms since preventing T cell priming and curbing fully-differentiated effectors may be fundamentally different processes.

Regarding the nature of the Treg, there has been much interest in recent years in the subdivision of Treg into subsets based largely on their transcription factor expression [119–123] or anatomical location [124,125]. Frequently the transcriptional profile of a given Treg subset is reported to parallel that of the effector T cell subset targeted for control [126]. While there are other ways to interpret these data [127], at face value the message is that Treg are not a homogenous population. It follows that different subsets of Treg could employ particular suppressive mechanisms to differing extents. The careful characterization of human Treg populations by the Sakaguchi laboratory revealed that those expressing CD45RA produce more TGFβ while the CD45RA-negative subset bear higher CTLA-4 expression and are capable of greater IL-10 production [128]. Thus, the type or differentiation state of the Treg population in question may influence the dominant mechanism of suppression it employs.

### 5.2 Does CTLA-4 work differently in Treg and Tconv – do CTLA-4-expressing Tconv have regulatory function?

If we accept that CTLA-4 can indeed function in both the Treg and Tconv compartments, one question that arises is how do these functions differ? Early models of CTLA-4 function were based on the notion that this receptor delivers a negative signal [101–103], and certainly many of my own papers were written from this perspective [105,129,130]. However, more recently the concept has emerged that CTLA-4 can function cell-extrinsically, indirectly controlling the responses of T cells that do not in fact express it [13]. This begs the question of whether CTLA-4 functions one way in conventional T cells (for example transducing negative signals to inhibit their activation) and another way in Treg (for example triggering ligand downregulation on antigen presenting cells [99,115]). Recent findings have shed new light on this issue; surprisingly, both the Allison laboratory [110] and my own group [111] found that conventional T cells were able to use CTLA-4 in a cell-extrinsic manner – essentially just like Treg do. Accordingly conventional T cells expressing CTLA-4 were able to regulate the proliferation of CTLA-4-deficient conventional T cells in their midst. Interestingly a microarray comparison of CTLA-4-sufficient and CTLA-4-deficient conventional T cells revealed “no obvious signature of active negative regulation” in the former [110], suggesting that even in conventional T cells, extrinsic mechanisms of CTLA4 function may be more important than the transmission of negative signals.

If CTLA-4 functions the same way in Treg and Tconv, and can direct a cell-extrinsic regulatory program, does this then blur the distinction between Tconv and Treg? In other words, are we now claiming that all T cells are essentially regulatory T cells by virtue of their ability to express CTLA-4? We recently explored this issue by comparing the capacity of CTLA-4-sufficient Tconv and CTLA-4-sufficient Treg to control the lymphoproliferative disease associated with CTLA-4 deficiency [111]. We took advantage of the fact that disease can be transferred into rag−/− recipients by injecting peripheral lymphocytes from CTLA-4−/− animals [131]. We found that even though co-transferred Tconv could express CTLA-4, they were unable to prevent disease, with the recipient animals losing as much weight as those receiving CTLA-4−/− cells alone. In contrast co-transferred Treg were highly effective at preventing disease and the recipient animals did not lose weight. This disease model is particularly aggressive, not least because the peripheral CTLA-4−/− lymphocytes are already activated at the point of adoptive transfer. To probe a little deeper we adapted the system by adoptively transferring CTLA-4−/− bone marrow rather than peripheral lymphocytes. This invokes a slower, less severe disease in the rag−/− recipients that we reasoned might be easier to control. Accordingly, adoptively transferred CTLA-4-sufficient Tconv were able to partially regulate disease in this setting leading to reduced weight loss, less severe tissue infiltration and decreased activation of peripheral T cells [111]. While this showed that Tconv-expressed CTLA-4 could partially regulate disease, it should be emphasized that regulation was modest compared to that invoked by CTLA-4-sufficient Treg. Recipients of the latter showed no weight loss (and instead gained weight), they lacked tissue infiltration and control of peripheral T cell activation was far more profound. Taken together, the overriding message is that although CTLA-4-sufficient Tconv exhibit modest suppressive capacity, Treg are far superior at eliciting regulation. This likely reflects their higher level of CTLA-4 expression and the fact that they express CTLA-4 constitutively, unlike Tconv that require a 2 day window to upregulate this protein [100,101,132]. Furthermore, the concommitant production of cytokines by conventional T cells, that are silenced in Treg by Foxp3, may serve to counteract suppression. Interestingly forced expression of CTLA-4 in activated T cells under the IL-2 promoter was able to significantly delay disease in CTLA-4-deficient mice [109]. Since IL-2 is induced rapidly upon T cell activation [133] early CTLA-4 induction may explain the superior capacity of these T cells to control disease, compared with Tconv that naturally express CTLA-4 in mice selectively lacking CTLA-4 in Treg [81]. The notion that Treg are the dominant population eliciting CTLA-4-dependent regulation, and that CTLA-4 function in Tconv plays a supplementary role, may explain the lack of complementation between scurfy and CTLA-4−/− bone marrow [82] since Treg may need to be present for the CTLA-4 effects on Tconv to be revealed.

## 6 Conclusion

**Fig. 2:**
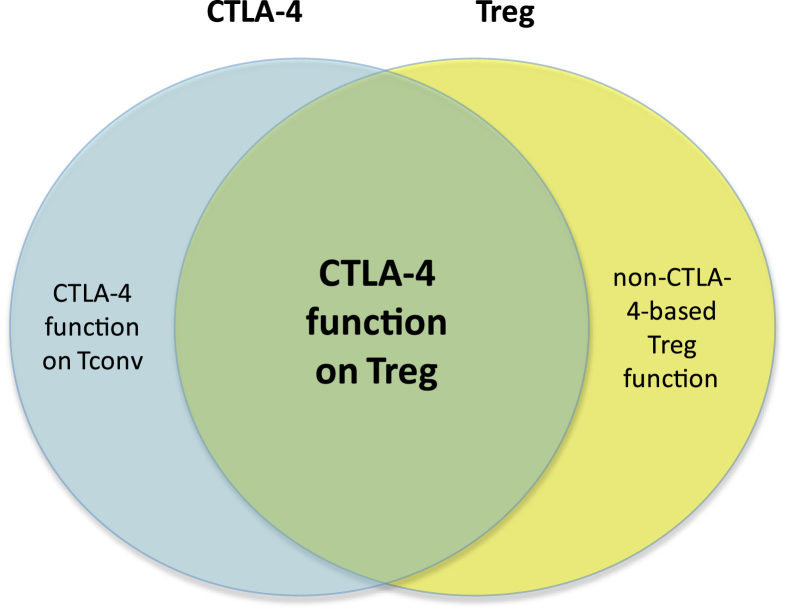
CTLA-4 and Foxp3 direct overlapping programs of immune regulation. A large fraction of CTLA-4-dependent immune regulation involves the actions of CTLA-4 on the Treg population; however CTLA-4 can also act on conventional T cells. Likewise, CTLA-4 represents a major mechanism of Treg function, but Treg can also call on numerous non-CTLA-4 based suppressive mechanisms.

Although the CTLA-4 and Foxp3 stories emerged independently of one another, they are nonetheless inextricably entwined (see Fig. 2). A large portion of CTLA-4-dependent immune regulation is achieved via expression of this molecule in the Treg compartment. Conversely Treg rely heavily, although by no means exclusively, on CTLA-4 to elicit regulatory function. Appreciation of the significant functional overlap between Foxp3 and CTLA-4 driven regulatory programs, and recognition of the cases where these pathways diverge, will guide the future harnessing of these pathways in therapeutic settings.

## 7 Final comments

This review was written to honor Professor Abul Abbas who has been a valued mentor and friend to me for over a decade. It is part of an issue which is devoted to Professor Abul Abbas and part of the Journal of Autoimmunity's recognition of truly distinguished immunologists who have contributed so much to our field; previous honorees have included Harry Moutsopoulos, Ian Mackay, Noel Rose, Chella David and Pierre Youinou [134–136]. My initial studies into both CTLA-4 and regulatory T cells emanated form work carried out in Abul's lab where I remember with great fondness my time as a Wellcome Trust postdoctoral fellow. His warmth, openness and generosity and his excitement over new data are things I recall vividly. Abul's ability to convey the essence of a seminar in a single sentence and his unwavering grasp on the “big picture” are things I will always admire. It's a pleasure to be able to offer this article in recognition of Abul's great contribution to the field of immunology.

## Figures and Tables

**Table 1:** The Foxp3−/− and CTLA-4−/− phenotypes can be corrected by the presence of wildtype cells. The effect of adoptive transfer of the indicated bone marrow into rag-deficient recipients, alone or with additional cells, is shown (in terms of whether recipients became sick or remained healthy). Note: Depending on the study, “Foxp3 deficient bone marrow” refers to bone marrow from mice lacking the Foxp3 gene or bone marrow from scurfy mice that have a frameshift mutation in the Foxp3 gene.

	Foxp3-deficient bone marrow	CTLA-4−/− bone marrow
Alone	Sick [82,83]	Sick [82,85,87,111]
Plus wildtype bone marrow	Healthy [82,83]	Healthy [33,82,85–88]
Plus wildtype CD4+CD25+ cells	Healthy [84]	Healthy [87,111]

**Table 2:** Examples of studies in which CTLA-4 has been shown to contribute to Treg function.

CTLA-4−/− Treg population tested	Observation	Reference
CD4+CD25+ cells	Regulation of colitis by CTLA-4-sufficient Treg was largely abrogated by anti-CTLA-4 blocking antibody. Note: CTLA-4−/− Treg were shown to regulate in an IL-10-dependent manner, suggesting other mechanisms can compensate in mice lacking CTLA-4 since birth.	[33]
Foxp3-expressing cells	Specific deletion of CTLA-4 in Treg (by expression of Foxp3-driven Cre in CTLA-4-floxed mice) caused lethal T cell mediated autoimmunity featuring lymphadenopathy, splenomegaly and widespread tissue infiltration.	[81]
CD4+CD25+ cells	DO11+CTLA4−/−rag−/− Treg were unable to control autoimmune pancreas destruction in an adoptive transfer model of diabetes. Wildtype Treg bearing an identical specificity (DO11+rag−/−) conferred 100% protection from disease.	[79]
CD4+CD25+ CD62^hi^ cells (from young Ctla4−/− mice)	CTLA-4−/− Treg failed to control colitis induced by T cell transfer into rag−/− recipients. Wildtype Treg conferred protection from colitis.	[94]
CD4+CD25+ cells	Injection of wildtype Treg but not conventional T cells (CD4+CD25−) significantly prolonged the lifespan of CTLA-4−/− mice.	[80]
CD4+CD25+ cells	Wildtype Treg completely prevented disease induced by CTLA-4−/− T cells in rag−/− recipients, demonstrating that expression of CTLA-4 in Treg is sufficient for regulation, even if conventional T cells lack CTLA-4.	[87]
CD4+CD25+ cells	Treg from CTLA-4−/− mice expressing CTLA-4 only in activated conventional T cells (under the control of the IL-2 promoter) failed to control colitis induced by CD4+CD25− cells in rag−/− recipients. Wildtype Treg conferred 100% protection.	[109]
CD4+CD25+ CD62^hi^ cells	CTLA-4+/+ but not CTLA-4−/− Treg reduced the infiltration of antigen-specific effector T cells into the pancreas and prevented the destruction of pancreatic tissue.	[108]
CD4+CD25+ cells (purified from healthy mixed bone marrow chimeras)	CTLA-4−/− Treg failed to suppress inflammatory bowel disease induced by T cell transfer into rag−/− recipients (0/4 mice survived). Recipients of wildtype Treg showed complete protection (4/4 mice survived).	[88]

## References

[1] Walker L.S., Abbas A.K. (2002). The enemy within: keeping self-reactive T cells at bay in the periphery. Nat Rev Immunol.

[2] Kim J.M., Rasmussen J.P., Rudensky A.Y. (2007). Regulatory T cells prevent catastrophic autoimmunity throughout the lifespan of mice. Nat Immunol.

[3] Tivol E.A., Borriello F., Schweitzer A.N., Lynch W.P., Bluestone J.A., Sharpe A.H. (1995). Loss of ctla-4 leads to massive lymphoproliferation and fatal multiorgan tissue destruction, revealing a critical negative regulatory role of ctla-4. Immunity.

[4] Waterhouse P., Penninger J.M., Timms E., Wakeham A., Shahinian A., Lee K.P. (1995). Lymphoproliferative disorders with early lethality in mice deficient in ctla-4. Science.

[5] Lenschow D.J., Walunas T.L., Bluestone J.A. (1996). CD28/B7 system of t cell costimulation. Annu Rev Immunol.

[6] Sansom D.M. (2000). Cd28, ctla-4 and their ligands: Who does what and to whom?. Immunology.

[7] Collins A.V., Brodie D.W., Gilbert R.J., Iaboni A., Manso-Sancho R., Walse B. (2002). The interaction properties of costimulatory molecules revisited. Immunity.

[8] Linsley P.S., Bradshaw J., Greene J., Peach R., Bennett K.L., Mittler R.S. (1996). Intracellular trafficking of ctla-4 and focal localisation towards sites of TCR engagement. Immunity.

[9] Teft W.A., Kirchhof M.G., Madrenas J. (2006). A molecular perspective of ctla-4 function. Annu Rev Immunol.

[10] Rudd C.E., Taylor A., Schneider H. (2009). CD28 and ctla-4 coreceptor expression and signal transduction. Immunol Rev.

[11] Bour-Jordan H., Esensten J.H., Martinez-Llordella M., Penaranda C., Stumpf M., Bluestone J.A. (2011). Intrinsic and extrinsic control of peripheral t-cell tolerance by costimulatory molecules of the CD28/B7 family. Immunol Rev.

[12] Wing K., Yamaguchi T., Sakaguchi S. (2011). Cell-autonomous and -non-autonomous roles of ctla-4 in immune regulation. Trends Immunol.

[13] Walker L.S., Sansom D.M. (2011). The emerging role of ctla4 as a cell-extrinsic regulator of T cell responses. Nat Rev Immunol.

[14] Gozalo-Sanmillan S., McNally J.M., Lin M.Y., Chambers C.A., Berg L.J. (2001). Cutting edge: two distinct mechanisms lead to impaired T cell homeostasis in janus kinase 3- and ctla-4-deficient mice. J Immunol.

[15] Chambers C.A., Sullivan T.J., Allison J.P. (1997). Lymphoproliferation in ctla-4-deficient mice is mediated by costimulation-dependent activation of cd4+ cells. Immunity.

[16] Karandikar N.J., Vanderlugt C.L., Walunas T.L., Miller S.D., Bluestone J.A. (1996). Ctla-4: a negative regulator of autoimmune disease. J Exp Med.

[17] Luhder F., Hoglund P., Allison J.P., Benoist C., Mathis D. (1998). Cytotoxic T lymphocyte-associated antigen 4 (ctla-4) regulates the unfolding of autoimmune diabetes. J Exp Med.

[18] Luhder F., Chambers C., Allison J.P., Benoist C., Mathis D. (2000). Pinpointing when T cell costimulatory receptor ctla-4 must be engaged to dampen diabetogenic T cells. Proc Natl Acad Sci U S A.

[19] Takahashi T., Tagami T., Yamazaki S., Uede T., Shimizu J., Sakaguchi N. (2000). Immunologic self-tolerance maintained by cd25(+)cd4(+) regulatory T cells constitutively expressing cytotoxic T lymphocyte-associated antigen 4. J Exp Med.

[20] Nistico L., Buzzetti R., Pritchard L.E., Van der Auwera B., Giovannini C., Bosi E. (1996). The ctla-4 gene region of chromosome 2q33 is linked to, and associated with, type 1 diabetes. Belgian diabetes registry. Hum Mol Genet.

[21] Ueda H., Howson J.M., Esposito L., Heward J., Snook H., Chamberlain G. (2003). Association of the T-cell regulatory gene ctla4 with susceptibility to autoimmune disease. Nature.

[22] Gough S.C., Walker L.S., Sansom D.M. (2005). Ctla4 gene polymorphism and autoimmunity. Immunol Rev.

[23] Butty V., Roy M., Sabeti P., Besse W., Benoist C., Mathis D. (2007). Signatures of strong population differentiation shape extended haplotypes across the human cd28, ctla4, and icos costimulatory genes. Proc Natl Acad Sci U S A.

[24] Oaks M.K., Hallett K.M. (2000). Cutting edge: a soluble form of ctla-4 in patients with autoimmune thyroid disease. J Immunol.

[25] Vijayakrishnan L., Slavik J.M., Illes Z., Greenwald R.J., Rainbow D., Greve B. (2004). An autoimmune disease-associated ctla-4 splice variant lacking the b7 binding domain signals negatively in T cells. Immunity.

[26] Araki M., Chung D., Liu S., Rainbow D.B., Chamberlain G., Garner V. (2009). Genetic evidence that the differential expression of the ligand-independent isoform of ctla-4 is the molecular basis of the idd5.1 type 1 diabetes region in nonobese diabetic mice. J Immunol.

[27] Gerold K.D., Zheng P., Rainbow D.B., Zernecke A., Wicker L.S., Kissler S. (2011). The soluble ctla-4 splice variant protects from type 1 diabetes and potentiates regulatory T-cell function. Diabetes.

[28] Liu S.M., Sutherland A.P., Zhang Z., Rainbow D.B., Quintana F.J., Paterson A.M. (2012). Overexpression of the ctla-4 isoform lacking exons 2 and 3 causes autoimmunity. J Immunol.

[29] Stumpf M., Zhou X., Bluestone J.A. (2013). The B7-independent isoform of ctla-4 functions to regulate autoimmune diabetes. J Immunol.

[30] Tivol E.A., Boyd S.D., McKeon S., Borriello F., Nickerson P., Strom T.B. (1997). Ctla4Ig prevents lymphoproliferation and fatal multiorgan tissue destruction in ctla-4-deficient mice. J Immunol.

[31] Tang Q., Boden E.K., Henriksen K.J., Bour-Jordan H., Bi M., Bluestone J.A. (2004). Distinct roles of ctla-4 and tgf-beta in cd4+cd25+ regulatory T cell function. Eur J Immunol.

[32] Mandelbrot D.A., McAdam A.J., Sharpe A.H. (1999). B7-1 or B7-2 is required to produce the lymphoproliferative phenotype in mice lacking cytotoxic t lymphocyte-associated antigen 4 (ctla-4). J Exp Med.

[33] Read S., Greenwald R., Izcue A., Robinson N., Mandelbrot D., Francisco L. (2006). Blockade of ctla-4 on cd4+cd25+ regulatory T cells abrogates their function in vivo. J Immunol.

[34] Mandelbrot D.A., Oosterwegel M.A., Shimizu K., Yamada A., Freeman G.J., Mitchell R.N. (2001). B7-dependent T-cell costimulation in mice lacking cd28 and ctla4. J Clin Invest.

[35] Tai X., Van Laethem F., Sharpe A.H., Singer A. (2007). Induction of autoimmune disease in ctla-4−/− mice depends on a specific CD28 motif that is required for in vivo costimulation. Proc Natl Acad Sci U S A.

[36] Powrie F., Mason D. (1990). Ox-22high CD4+ T cells induce wasting disease with multiple organ pathology: prevention by the ox-22low subset. J Exp Med.

[37] Read S., Mauze S., Asseman C., Bean A., Coffman R., Powrie F. (1998). Cd38+ cd45rb(low) CD4+ T cells: a population of T cells with immune regulatory activities in vitro. Eur J Immunol.

[38] Sakaguchi S., Sakaguchi N., Asano M., Itoh M., Toda M. (1995). Immunologic self-tolerance maintained by activated T cells expressing il-2 receptor alpha-chains (cd25). Breakdown of a single mechanism of self-tolerance causes various autoimmune diseases. J Immunol.

[39] Asano M., Toda M., Sakaguchi N., Sakaguchi S. (1996). Autoimmune disease as a consequence of developmental abnormality of a T cell subpopulation. J Exp Med.

[40] Suri-Payer E., Amar A.Z., Thornton A.M., Shevach E.M. (1998). Cd4+cd25+ T cells inhibit both the induction and effector function of autoreactive T cells and represent a unique lineage of immunoregulatory cells. J Immunol.

[41] Thornton A.M., Shevach E.M. (1998). Cd4+cd25+ immunoregulatory T cells suppress polyclonal T cell activation in vitro by inhibiting interleukin 2 production. J Exp Med.

[42] Sakaguchi S., Sakaguchi N., Shimizu J., Yamazaki S., Sakihama T., Itoh M. (2001). Immunologic tolerance maintained by cd25+ cd4+ regulatory T cells: their common role in controlling autoimmunity, tumor immunity, and transplantation tolerance. Immunol Rev.

[43] Shevach E.M., DiPaolo R.A., Andersson J., Zhao D.M., Stephens G.L., Thornton A.M. (2006). The lifestyle of naturally occurring cd4+ cd25+ foxp3+ regulatory T cells. Immunol Rev.

[44] Bacchetta R., Gambineri E., Roncarolo M.G. (2007). Role of regulatory T cells and foxp3 in human diseases. J Allergy Clin Immunol.

[45] Somerset D.A., Zheng Y., Kilby M.D., Sansom D.M., Drayson M.T. (2004). Normal human pregnancy is associated with an elevation in the immune suppressive cd25+ cd4+ regulatory t-cell subset. Immunology.

[46] Zenclussen A.C., Gerlof K., Zenclussen M.L., Sollwedel A., Bertoja A.Z., Ritter T. (2005). Abnormal t-cell reactivity against paternal antigens in spontaneous abortion: adoptive transfer of pregnancy-induced cd4+cd25+ t regulatory cells prevents fetal rejection in a murine abortion model. Am J Pathol.

[47] Aluvihare V.R., Kallikourdis M., Betz A.G. (2004). Regulatory T cells mediate maternal tolerance to the fetus. Nat Immunol.

[48] Shima T., Sasaki Y., Itoh M., Nakashima A., Ishii N., Sugamura K. (2010). Regulatory T cells are necessary for implantation and maintenance of early pregnancy but not late pregnancy in allogeneic mice. J Reprod Immunol.

[49] Samstein R.M., Josefowicz S.Z., Arvey A., Treuting P.M., Rudensky A.Y. (2012). Extrathymic generation of regulatory T cells in placental mammals mitigates maternal-fetal conflict. Cell.

[50] Vignali D.A., Collison L.W., Workman C.J. (2008). How regulatory T cells work. Nat Rev Immunol.

[51] Tang Q., Bluestone J.A. (2008). The foxp3+ regulatory T cell: a jack of all trades, master of regulation. Nat Immunol.

[52] Brunkow M.E., Jeffery E.W., Hjerrild K.A., Paeper B., Clark L.B., Yasayko S.A. (2001). Disruption of a new forkhead/winged-helix protein, scurfin, results in the fatal lymphoproliferative disorder of the scurfy mouse. Nat Genet.

[53] Godfrey V.L., Wilkinson J.E., Russell L.B. (1991). X-linked lymphoreticular disease in the scurfy (sf) mutant mouse. Am J Pathol.

[54] Hori S., Nomura T., Sakaguchi S. (2003). Control of regulatory T cell development by the transcription factor foxp3. Science.

[55] Fontenot J.D., Gavin M.A., Rudensky A.Y. (2003). Foxp3 programs the development and function of cd4+cd25+ regulatory T cells. Nat Immunol.

[56] Khattri R., Cox T., Yasayko S.A., Ramsdell F. (2003). An essential role for scurfin in cd4+cd25+ t regulatory cells. Nat Immunol.

[57] Wildin R.S., Ramsdell F., Peake J., Faravelli F., Casanova J.L., Buist N. (2001). X-linked neonatal diabetes mellitus, enteropathy and endocrinopathy syndrome is the human equivalent of mouse scurfy. Nat Genet.

[58] Bennett C.L., Christie J., Ramsdell F., Brunkow M.E., Ferguson P.J., Whitesell L. (2001). The immune dysregulation, polyendocrinopathy, enteropathy, x-linked syndrome (ipex) is caused by mutations of foxp3. Nat Genet.

[59] Gavin M.A., Rasmussen J.P., Fontenot J.D., Vasta V., Manganiello V.C., Beavo J.A. (2007). Foxp3-dependent programme of regulatory t-cell differentiation. Nature.

[60] Ohkura N., Hamaguchi M., Morikawa H., Sugimura K., Tanaka A., Ito Y. (2012). T cell receptor stimulation-induced epigenetic changes and foxp3 expression are independent and complementary events required for treg cell development. Immunity.

[61] Fontenot J.D., Dooley J.L., Farr A.G., Rudensky A.Y. (2005). Developmental regulation of foxp3 expression during ontogeny. J Exp Med.

[62] Sekiya T., Kashiwagi I., Yoshida R., Fukaya T., Morita R., Kimura A. (2013). Nr4a receptors are essential for thymic regulatory T cell development and immune homeostasis. Nat Immunol.

[63] Tai X., Cowan M., Feigenbaum L., Singer A. (2005). Cd28 costimulation of developing thymocytes induces foxp3 expression and regulatory T cell differentiation independently of interleukin 2. Nat Immunol.

[64] Khattri R., Kasprowicz D., Cox T., Mortrud M., Appleby M.W., Brunkow M.E. (2001). The amount of scurfin protein determines peripheral T cell number and responsiveness. J Immunol.

[65] Walker L.S., Chodos A., Eggena M., Dooms H., Abbas A.K. (2003). Antigen-dependent proliferation of cd4+ cd25+ regulatory T cells in vivo. J Exp Med.

[66] Lathrop S.K., Santacruz N.A., Pham D., Luo J., Hsieh C.S. (2008). Antigen-specific peripheral shaping of the natural regulatory T cell population. J Exp Med.

[67] Zheng Y., Josefowicz S.Z., Kas A., Chu T.T., Gavin M.A., Rudensky A.Y. (2007). Genome-wide analysis of foxp3 target genes in developing and mature regulatory T cells. Nature.

[68] Marson A., Kretschmer K., Frampton G.M., Jacobsen E.S., Polansky J.K., MacIsaac K.D. (2007). Foxp3 occupancy and regulation of key target genes during t-cell stimulation. Nature.

[69] Rudra D., deRoos P., Chaudhry A., Niec R.E., Arvey A., Samstein R.M. (2012). Transcription factor foxp3 and its protein partners form a complex regulatory network. Nat Immunol.

[70] Wu Y., Borde M., Heissmeyer V., Feuerer M., Lapan A.D., Stroud J.C. (2006). Foxp3 controls regulatory T cell function through cooperation with nfat. Cell.

[71] Ono M., Yaguchi H., Ohkura N., Kitabayashi I., Nagamura Y., Nomura T. (2007). Foxp3 controls regulatory t-cell function by interacting with aml1/runx1. Nature.

[72] Zheng Y., Chaudhry A., Kas A., deRoos P., Kim J.M., Chu T.T. (2009). Regulatory t-cell suppressor program co-opts transcription factor irf4 to control t(h)2 responses. Nature.

[73] Dang E.V., Barbi J., Yang H.Y., Jinasena D., Yu H., Zheng Y. (2011). Control of t(h)17/t(reg) balance by hypoxia-inducible factor 1. Cell.

[74] Allan S.E., Passerini L., Bacchetta R., Crellin N., Dai M., Orban P.C. (2005). The role of 2 foxp3 isoforms in the generation of human cd4+ tregs. J Clin Invest.

[75] Zheng Y., Manzotti C.N., Burke F., Dussably L., Qureshi O., Walker L.S. (2008). Acquisition of suppressive function by activated human cd4+ cd25− T cells is associated with the expression of ctla-4 not foxp3. J Immunol.

[76] Fu W., Ergun A., Lu T., Hill J.A., Haxhinasto S., Fassett M.S. (2012). A multiply redundant genetic switch ‘locks in’ the transcriptional signature of regulatory T cells. Nat Immunol.

[77] Huang C., Martin S., Pfleger C., Du J., Buckner J.H., Bluestone J.A. (2013). Cutting edge: a novel, human-specific interacting protein couples foxp3 to a chromatin-remodeling complex that contains kap1/trim28. J Immunol.

[78] Metzler B., Burkhart C., Wraith D.C. (1999). Phenotypic analysis of ctla-4 and cd28 expression during transient peptide-induced T cell activation in vivo. Int Immunol.

[79] Schmidt E.M., Wang C.J., Ryan G.A., Clough L.E., Qureshi O.S., Goodall M. (2009). Ctla-4 controls regulatory T cell peripheral homeostasis and is required for suppression of pancreatic islet autoimmunity. J Immunol.

[80] Kolar P., Knieke K., Hegel J.K., Quandt D., Burmester G.R., Hoff H. (2009). Ctla-4 (cd152) controls homeostasis and suppressive capacity of regulatory T cells in mice. Arthritis Rheum.

[81] Wing K., Onishi Y., Prieto-Martin P., Yamaguchi T., Miyara M., Fehervari Z. (2008). Ctla-4 control over foxp3+ regulatory T cell function. Science.

[82] Chikuma S., Bluestone J.A. (2007). Expression of ctla-4 and foxp3 in cis protects from lethal lymphoproliferative disease. Eur J Immunol.

[83] Ouyang W., Beckett O., Ma Q., Paik J.H., DePinho R.A., Li M.O. (2010). Foxo proteins cooperatively control the differentiation of foxp3+ regulatory T cells. Nat Immunol.

[84] Komatsu N., Hori S. (2007). Full restoration of peripheral foxp3+ regulatory T cell pool by radioresistant host cells in scurfy bone marrow chimeras. Proc Natl Acad Sci U S A.

[85] Bachmann M.F., Kohler G., Ecabert B., Mak T.W., Kopf M. (1999). Cutting edge: lymphoproliferative disease in the absence of ctla-4 is not T cell autonomous. J Immunol.

[86] Homann D., Dummer W., Wolfe T., Rodrigo E., Theofilopoulos A.N., Oldstone M.B. (2006). Lack of intrinsic ctla-4 expression has minimal effect on regulation of antiviral t-cell immunity. J Virol.

[87] Friedline R.H., Brown D.S., Nguyen H., Kornfeld H., Lee J., Zhang Y. (2009). Cd4+ regulatory T cells require ctla-4 for the maintenance of systemic tolerance. J Exp Med.

[88] Tai X., Van Laethem F., Pobezinsky L., Guinter T., Sharrow S.O., Adams A. (2012). Basis of ctla-4 function in regulatory and conventional cd4(+) T cells. Blood.

[89] Read S., Malmstrom V., Powrie F. (2000). Cytotoxic t lymphocyte-associated antigen 4 plays an essential role in the function of cd25(+)cd4(+) regulatory cells that control intestinal inflammation. J Exp Med.

[90] Chai J.G., Tsang J.Y., Lechler R., Simpson E., Dyson J., Scott D. (2002). Cd4+cd25+ T cells as immunoregulatory T cells in vitro. Eur J Immunol.

[91] Thornton A.M., Piccirillo C.A., Shevach E.M. (2004). Activation requirements for the induction of cd4+cd25+ T cell suppressor function. Eur J Immunol.

[92] Verhagen J., Gabrysova L., Minaee S., Sabatos C.A., Anderson G., Sharpe A.H. (2009). Enhanced selection of foxp3+ t-regulatory cells protects ctla-4-deficient mice from cns autoimmune disease. Proc Natl Acad Sci U S A.

[93] Kataoka H., Takahashi S., Takase K., Yamasaki S., Yokosuka T., Koike T. (2005). Cd25(+)cd4(+) regulatory T cells exert in vitro suppressive activity independent of ctla-4. Int Immunol.

[94] Sojka D.K., Hughson A., Fowell D.J. (2009). Ctla-4 is required by cd4+cd25+ treg to control cd4+ t-cell lymphopenia-induced proliferation. Eur J Immunol.

[95] Pillai M.R., Collison L.W., Wang X., Finkelstein D., Rehg J.E., Boyd K. (2011). The plasticity of regulatory T cell function. J Immunol.

[96] Jonuleit H., Schmitt E., Stassen M., Tuettenberg A., Knop J., Enk A.H. (2001). Identification and functional characterization of human cd4(+)cd25(+) T cells with regulatory properties isolated from peripheral blood. J Exp Med.

[97] Annunziato F., Cosmi L., Liotta F., Lazzeri E., Manetti R., Vanini V. (2002). Phenotype, localization, and mechanism of suppression of cd4(+)cd25(+) human thymocytes. J Exp Med.

[98] Manzotti C.N., Tipping H., Perry L.C., Mead K.I., Blair P.J., Zheng Y. (2002). Inhibition of human T cell proliferation by ctla-4 utilizes cd80 and requires cd25+ regulatory T cells. Eur J Immunol.

[99] Qureshi O.S., Zheng Y., Nakamura K., Attridge K., Manzotti C., Schmidt E.M. (2011). Trans-endocytosis of cd80 and cd86: a molecular basis for the cell-extrinsic function of ctla-4. Science.

[100] Walunas T.L., Lenschow D.J., Bakker C.Y., Linsley P.S., Freeman G.J., Green J.M. (1994). Ctla-4 can function as a negative regulator of T cell activation. Immunity.

[101] Krummel M.F., Allison J.P. (1995). Cd28 and ctla-4 have opposing effects on the response of T cells to stimulation. J Exp Med.

[102] Krummel M.F., Allison J.P. (1996). Ctla-4 engagement inhibits il-2 accumulation and cell cycle progression upon activation of resting T cells. J Exp Med.

[103] Walunas T.L., Bakker C.Y., Bluestone J.A. (1996). Ctla-4 ligation blocks cd28-dependent T cell activation. J Exp Med.

[104] Greenwald R.J., Boussiotis V.A., Lorsbach R.B., Abbas A.K., Sharpe A.H. (2001). Ctla-4 regulates induction of anergy in vivo. Immunity.

[105] Walker L.S., Ausubel L.J., Chodos A., Bekarian N., Abbas A.K. (2002). Ctla-4 differentially regulates T cell responses to endogenous tissue protein versus exogenous immunogen. J Immunol.

[106] Eggena M.P., Walker L.S., Nagabhushanam V., Barron L., Chodos A., Abbas A.K. (2004). Cooperative roles of ctla-4 and regulatory T cells in tolerance to an islet cell antigen. J Exp Med.

[107] Peggs K.S., Quezada S.A., Chambers C.A., Korman A.J., Allison J.P. (2009). Blockade of ctla-4 on both effector and regulatory T cell compartments contributes to the antitumor activity of anti-ctla-4 antibodies. J Exp Med.

[108] Ise W., Kohyama M., Nutsch K.M., Lee H.M., Suri A., Unanue E.R. (2010). Ctla-4 suppresses the pathogenicity of self antigen-specific T cells by cell-intrinsic and cell-extrinsic mechanisms. Nat Immunol.

[109] Jain N., Nguyen H., Chambers C., Kang J. (2010). Dual function of ctla-4 in regulatory T cells and conventional T cells to prevent multiorgan autoimmunity. Proc Natl Acad Sci U S A.

[110] Corse E., Allison J.P. (2012). Cutting edge: ctla-4 on effector T cells inhibits in trans. J Immunol.

[111] Wang C.J., Kenefeck R., Wardzinski L., Attridge K., Manzotti C., Schmidt E.M. (2012). Cutting edge: cell-extrinsic immune regulation by ctla-4 expressed on conventional T cells. J Immunol.

[112] Thompson C.B., Allison J.P. (1997). The emerging role of ctla-4 as an immune attenuator. Immunity.

[113] Cederbom L., Hall H., Ivars F. (2000). Cd4+cd25+ regulatory T cells down-regulate co-stimulatory molecules on antigen-presenting cells. Eur J Immunol.

[114] Oderup C., Cederbom L., Makowska A., Cilio C.M., Ivars F. (2006). Cytotoxic t lymphocyte antigen-4-dependent down-modulation of costimulatory molecules on dendritic cells in cd4+ cd25+ regulatory t-cell-mediated suppression. Immunology.

[115] Onishi Y., Fehervari Z., Yamaguchi T., Sakaguchi S. (2008). Foxp3+ natural regulatory T cells preferentially form aggregates on dendritic cells in vitro and actively inhibit their maturation. Proc Natl Acad Sci U S A.

[116] Pennline K.J., Roque-Gaffney E., Monahan M. (1994). Recombinant human il-10 prevents the onset of diabetes in the nonobese diabetic mouse. Clin Immunol Immunopathol.

[117] Phillips J.M., Parish N.M., Drage M., Cooke A. (2001). Cutting edge: interactions through the il-10 receptor regulate autoimmune diabetes. J Immunol.

[118] Wogensen L., Lee M.S., Sarvetnick N. (1994). Production of interleukin 10 by islet cells accelerates immune-mediated destruction of beta cells in nonobese diabetic mice. J Exp Med.

[119] Koch M.A., Tucker-Heard G., Perdue N.R., Killebrew J.R., Urdahl K.B., Campbell D.J. (2009). The transcription factor t-bet controls regulatory T cell homeostasis and function during type 1 inflammation. Nat Immunol.

[120] Chaudhry A., Rudra D., Treuting P., Samstein R.M., Liang Y., Kas A. (2009). Cd4+ regulatory T cells control th17 responses in a stat3-dependent manner. Science.

[121] Chung Y., Tanaka S., Chu F., Nurieva R.I., Martinez G.J., Rawal S. (2011). Follicular regulatory T cells expressing foxp3 and bcl-6 suppress germinal center reactions. Nat Med.

[122] Linterman M.A., Pierson W., Lee S.K., Kallies A., Kawamoto S., Rayner T.F. (2011). Foxp3+ follicular regulatory T cells control the germinal center response. Nat Med.

[123] Wollenberg I., Agua-Doce A., Hernandez A., Almeida C., Oliveira V.G., Faro J. (2011). Regulation of the germinal center reaction by foxp3+ follicular regulatory T cells. J Immunol.

[124] Feuerer M., Herrero L., Cipolletta D., Naaz A., Wong J., Nayer A. (2009). Lean, but not obese, fat is enriched for a unique population of regulatory T cells that affect metabolic parameters. Nat Med.

[125] Cipolletta D., Feuerer M., Li A., Kamei N., Lee J., Shoelson S.E. (2012). Ppar-gamma is a major driver of the accumulation and phenotype of adipose tissue treg cells. Nature.

[126] Barnes M.J., Powrie F. (2009). Hybrid treg cells: steel frames and plastic exteriors. Nat Immunol.

[127] Tian L., Humblet-Baron S., Liston A. (2012). Immune tolerance: are regulatory T cell subsets needed to explain suppression of autoimmunity?. Bioessays.

[128] Miyara M., Yoshioka Y., Kitoh A., Shima T., Wing K., Niwa A. (2009). Functional delineation and differentiation dynamics of human cd4+ T cells expressing the foxp3 transcription factor. Immunity.

[129] Walker L.S., Wiggett H.E., Gaspal F.M., Raykundalia C.R., Goodall M.D., Toellner K.M. (2003). Established T cell-driven germinal center b cell proliferation is independent of cd28 signaling but is tightly regulated through ctla-4. J Immunol.

[130] Sansom D.M., Walker L.S. (2006). The role of cd28 and cytotoxic t-lymphocyte antigen-4 (ctla-4) in regulatory t-cell biology. Immunol Rev.

[131] Tivol E.A., Gorski J. (2002). Re-establishing peripheral tolerance in the absence of ctla-4: complementation by wild-type T cells points to an indirect role for ctla-4. J Immunol.

[132] Linsley P.S., Greene J.L., Tan P., Bradshaw J., Ledbetter J.A., Anasetti C. (1992). Coexpression and functional cooperation of ctla-4 and cd28 on activated t lymphocytes. J Exp Med.

[133] Sojka D.K., Bruniquel D., Schwartz R.H., Singh N.J. (2004). Il-2 secretion by cd4+ T cells in vivo is rapid, transient, and influenced by tcr-specific competition. J Immunol.

[134] Gershwin M.E., Shoenfeld Y. (2011). Chella David: a lifetime contribution in translational immunology. J Autoimmun.

[135] Jamin C., Renaudineau Y., Pers J.O. (2012). Pierre Youinou: when intuition and determination meet autoimmunity. J Autoimmun.

[136] Tzioufas A.G., Kapsogeorgou E.K., Moutsopoulos H.M. (2012). Pathogenesis of Sjogren's syndrome: what we know and what we should learn. J Autoimmun.

